# Breast milk–associated physiological hypercalcaemia: an observational study of clinical, biochemical and radiological outcomes

**DOI:** 10.1136/bmjpo-2025-004230

**Published:** 2026-05-28

**Authors:** Sandhya Govindarajan, Mohamed Zulf Mughal, Imran Zamir, Raja Padidela, Amish Chinoy

**Affiliations:** 1 Manchester University NHS Foundation Trust, Manchester, UK; 2 Al Jalila Children’s Specialty Hospital, Dubai, Dubai, UAE; 3 Royal Manchester Children’s Hospital, Manchester, UK; 4 Department of Paediatric Endocrinology, Royal Manchester Children’s Hospital, Manchester, UK; 5 The University of Manchester Faculty of Biology Medicine and Health, Manchester, UK

**Keywords:** Endocrinology, Neonatology

## Abstract

**Introduction:**

Asymptomatic physiological hypercalcaemia in exclusively breastfed infants is recognised but has not been systematically characterised when breastfeeding is continued.

**Objective:**

To evaluate the clinical, biochemical and radiological outcomes of breast milk–associated physiological hypercalcaemia.

**Methods:**

A multi-centre retrospective study over 5 years included infants with hypercalcaemia (serum corrected calcium (cCa) ≥2.8 mmol/L) who were exclusively breastfed and had no other identifiable causes of hypercalcaemia. Clinical, biochemical and radiological data were analysed.

**Results:**

Twenty-five infants (15 males, 10 females) were studied. The mean peak cCa was 3.08 mmol/L (SD 0.17; normal 2.2–2.8 mmol/L). All infants were asymptomatic and continued exclusive breastfeeding without interventions such as intravenous fluids, diuretics or bisphosphonates. Mean serum parathyroid hormone (PTH) was suppressed at 1.04 pmol/L (SD 0.9; reference 2.0–9.4 pmol/L). Renal ultrasound examinations performed in all infants showed no nephrocalcinosis. Hypercalcaemia resolved spontaneously over a median of 64 (range, 9–329) days, and the median duration of exclusive breastfeeding was 150 (range, 8–540) days.

**Conclusion:**

Breast milk–associated physiological hypercalcaemia is a benign, PTH-independent condition without clinical symptoms or nephrocalcinosis. Infants can safely continue exclusive breastfeeding, preserving its well-established benefits, without switching to low calcium formulas. Further research is needed to elucidate the underlying mechanisms.

**WHAT IS ALREADY KNOWN ON THIS TOPIC**

- Physiological hypercalcaemia has been reported in breastfed infants, but uncertainty about its natural history often leads to intervention or cessation of breastfeeding.

**WHAT THIS STUDY ADDS**

- This study shows that this form of hypercalcaemia follows a self-limiting course with suppressed parathyroid hormone and no evidence of renal complications based on serial biochemical and imaging follow-up.

**HOW THIS STUDY MIGHT AFFECT RESEARCH, PRACTICE OR POLICY**

- These findings support a conservative approach with continued breastfeeding and serial monitoring, reducing unnecessary treatments and reinforcing breastfeeding as safe in this context.

## Introduction

 International recommendations are that infants should receive breast milk for the first 6 months of life, with many advantages for the infant and mother.^1 2^ The main benefit is protection against gastrointestinal infections (in both developing and developed countries), and it reduces the risk of mortality due to diarrhoea.^1^ Breastfed children have reduced incidence of obesity, perform better on intelligence tests, have higher school attendance and higher income in adult life. Breastfeeding also improves the well-being of mothers by reducing the risk of ovarian and breast cancer and helps space pregnancies.^2^

Hypercalcaemia in exclusively breastfed infants is relatively uncommon but can occur. Clinical features of hypercalcaemia in infancy are hypotonia, poor feeding, vomiting, constipation, failure to thrive, abdominal pain, polyuria, lethargy and dehydration.^3^ In severe cases, renal failure, pancreatitis and reduced consciousness can also occur.^3^ Measures such as discontinuation of breastfeeding, changing to low-calcium (Ca) formula, phosphate supplementation, treatment with normal saline and diuretics are sometimes adopted to lower serum Ca levels,^4^ due to concerns about the evolution of nephrocalcinosis and other systemic manifestations of hypercalcaemia.

However, the natural history and outcome of this phenomenon, in which it was continued, have never been described. Therefore, this study retrospectively explored the biochemical, radiological and clinical features and outcomes of hypercalcaemia in exclusively breastfed infants to understand the natural history and elucidate whether continuation of breast milk consumption remains safe in the context of hypercalcaemia, given its many other benefits.

## Methods

Infants aged ≤6 months referred to the tertiary paediatric metabolic bone disorders service at Royal Manchester Children’s Hospital over 5 years (January 2019 to December 2023) who had hypercalcaemia (serum corrected Ca (cCa) ≥ 2.8 mmol/L), were exclusively breastfed and were managed conservatively (ie, with monitoring, without dietary or medical interventions) were included. These infants had been admitted to hospital for other medical reasons (jaundice or feeding concerns being the the most common), in whom high Ca levels were detected incidentally as a part of routine investigations. Baseline data were collected retrospectively from seven hospitals across North-West England—Royal Manchester Children’s Hospital, North Manchester General Hospital, Royal Bolton Hospital, Royal Albert Edward Infirmary (Wigan), Tameside General Hospital, Wythenshawe Hospital and Royal Blackburn Hospital—using a standardised proforma to minimise observer bias, recall bias and inconsistencies in data recording. Clinical, radiological and biochemical data were collected from patient’s records. Infants with other medical conditions causing hypercalcaemia, formula-fed infants or mixed-fed infants, as well as preterm infants who received parenteral nutrition or breast milk fortification, were excluded from the study.

The sample size was determined by the total number of eligible cases available during the study period across participating hospitals. No formal sample size calculation was performed.

Longitudinal monitoring for the study commenced when exclusively breast-fed infants with hypercalcaemia were referred to the tertiary paediatric metabolic bone disorders centre. The same investigations were performed at all sites, although the timing of these assessments varied slightly and all data were collected using a standardised proforma to ensure consistency. These infants were prospectively followed up until the resolution of hypercalcaemia. Serum cCa levels were monitored every 8–12 weeks until normalisation.

## Results

Thirty infants with hypercalcaemia were initially identified, with five excluded because of loss to follow-up (3), mixed feeding (1) and other medical causes of hypercalcaemia (1). The final cohort comprised 25 infants (15 males, 10 females), including five preterm infants (<37 weeks of gestational age). All were clinically asymptomatic in the context of hypercalcaemia, and high cCa (≥2.8 mmol/L) was incidentally detected during routine laboratory testing.

Supporting data are provided in online supplemental table 1.

Table 1 summarises the biochemical profile of the cohort, showing appropriately suppressed mean parathyroid hormone (PTH), normal phosphate, 25-hydroxyvitamin D and magnesium and elevated spot urine Ca-to-creatinine ratio. *Importantly, all 25 infants underwent renal ultrasound imaging after detection of* *hypercalcaemia,* *and despite elevated urinary Ca, none of the infants developed nephrocalcinosis* supporting the benign nature of breast milk–associated physiological hypercalcaemia (see footnote).

**Table 1:** Bone biochemical parameters of 25 exclusively breastfed infants with hypercalcaemia

Variable	Number†	Mean (SD)	Median	Range (min–max)	Reference range
Peak corrected calcium, mmol/L	25	3.08 (0.17)	3.05	2.84–2.46	2.2–2.8
Corresponding 25-hydroxy vitamin D, nmol/L	21	59.7 (35.1)	48	18.9–141	>50
Corresponding parathyroid hormone, pmol/L	25	1.04 (0.88)	0.6	0.3–3.5	2.0–9.4
Corresponding phosphate, mmol/L	24	1.90 (0.40)	2	0.89–2.91	1.3–2.6
Corresponding magnesium, mmol/L	14	0.85 (0.11)	0.80	0.67–1.05	0.70–1.0
Corresponding spot urinary calcium-to-creatinine ratio, mmol/mmol	13	3.76 (2.97)	3.33	0.75–11.7	0.01–0.52*

Data are presented as mean (SD) or median (range) where indicated.

* Reference ranges shown are for adults; infant reference ranges are not well-defined and are generally higher.^13^

† Number of infants with available data is indicated for each variable.

**Figure 1:**
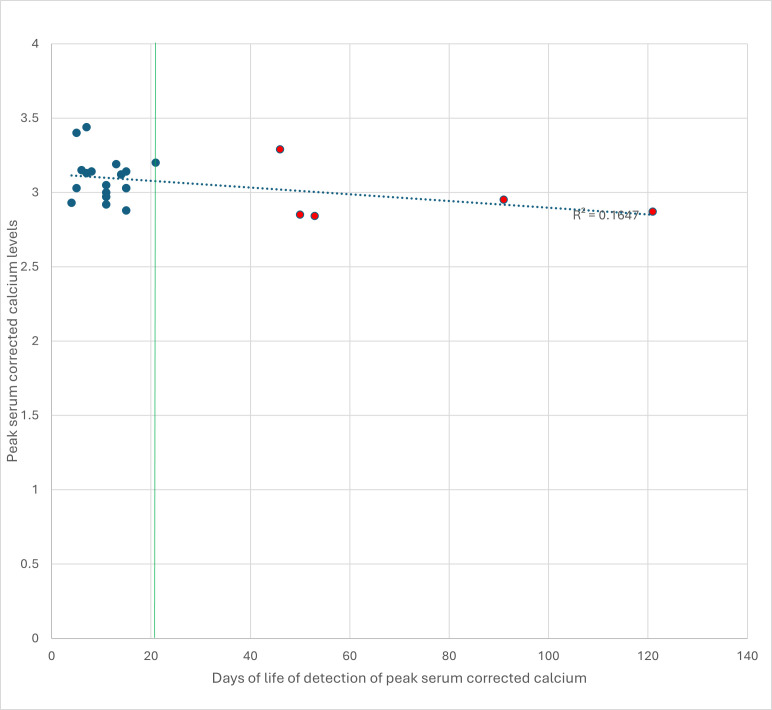
Graph showing days of life of detection of peak serum corrected calcium vs peak serum corrected calcium levels.

Figure 1 shows a scatter plot of days of life at peak serum cCa versus peak Ca levels in the 25 infants. The vertical green line at 21 days indicates the cut-off between early and late peak Ca. Twenty infants had a peak within the first 21 days, while five had a peak after day 20 (highlighted in red). A trendline demonstrates a very weak relationship (R² = 0.165), indicating that the day of life explained little of the variation in peak Ca.

Table 2 shows the timelines for detection and resolution of hypercalcaemia and duration of exclusive breastfeeding.

**Table 2:** Timeline and duration of hypercalcaemia and exclusive breastfeeding in 25 exclusively breastfed infants

Variable	Number (n)*	Median (X̃)	Range (min–max)
Day of life of detection of hypercalcaemia	24	14	(4–121)
Day of life of earliest renal ultrasound scan	19	17	(4–82)
Duration of exclusive breastfeeding in days	17	150	(8–540)
Day of life of resolution of hypercalcaemia	22	64	(9–329)
Total duration of hypercalcaemia in days	22	64	(1–208)

Median and range (min–max) are presented. Missing data reflect incomplete records in this retrospective cohort.

* (n)= number of infants with available data for the variable.

## Discussion

This retrospective study suggests that hypercalcaemia associated with breast milk consumption is PTH-independent, asymptomatic, does not cause nephrocalcinosis with continued breastfeeding, resolves spontaneously without intervention and hence can be considered physiological. We suggest referring to this condition as ‘breast milk–associated physiological hypercalcaemia’.

### Comparison with previous literature

Hypercalcaemia in exclusively breastfed infants is understudied. In 1981, Greer *et al* assessed 18-term infants and discussed ‘physiological hypercalcaemia’ in exclusively breastfed infants followed up for 6 months.^5^ This study found that breast milk phosphate decreased from 6 weeks to 6 months (p<0.001), leading to lower serum phosphate and higher Ca and magnesium levels independent of PTH. The present study also showed PTH-independent hypercalcaemia in exclusively breastfed infants, although we did not observe any abnormalities in serum phosphate in these infants.

PTH-related peptide (PTHrP) is produced in breast milk by lactating mothers.^6^ Although this could theoretically affect serum Ca levels in these infants, resulting in hypercalcaemia, previous studies have not demonstrated such an effect, suggesting that ingested PTHrP may act locally in the gut or be largely degraded during digestion rather than significantly affecting systemic Ca homeostasis.^7^

Transient neonatal hypercalcaemia from maternal hypoparathyroidism during pregnancy, associated with chronic maternal hypocalcaemia, usually presents with elevated PTH and skeletal demineralisation, occasionally resulting in fractures.^8^ Our study findings are in contrast to this highlighting that breast milk–associated physiological hypercalcaemia is a benign and distinct entity.

Idiopathic infantile hypercalcaemia (IIH) has a high risk of nephrocalcinosis. Lenherr-Taube *et al*^9^ (part 1) characterised the biochemical and genetic features of mild IIH and reported a *55% prevalence of nephrocalcinosis* in their cohort of affected children. Their findings underscore the importance of genetic testing in suspected IIH. Although routine genetic testing was not performed in our cohort, the overall clinical picture—PTH-independent hypercalcaemia, complete absence of symptoms and absence of *nephrocalcinosis in any infant*—makes a genetically mediated IIH phenotype unlikely.

In part 2 of the study,^10^ a longitudinal cohort demonstrated that *persistent hypercalcaemia was observed in 18%* of infants, even with ongoing dietary restriction. Although dietary modification improved serum and urinary Ca levels in all participants, complete normalisation was not consistently achieved, and some experienced recurrence of hypercalcaemia after reintroducing a normal diet in part 2 of the study. Together, these findings contrast sharply with our cohort, in which all infants were exclusively breastfed, remained asymptomatic, had *no nephrocalcinosis*, and showed *spontaneous resolution* of hypercalcaemia without dietary intervention. This comparison reinforces that breast milk–associated physiological hypercalcaemia is a benign, self-limiting condition distinct from IIH, supporting a different natural history.

### Potential mechanisms

Exact mechanism of hypercalcaemia in exclusively breastfed babies is unknown.

**Figure 2:**
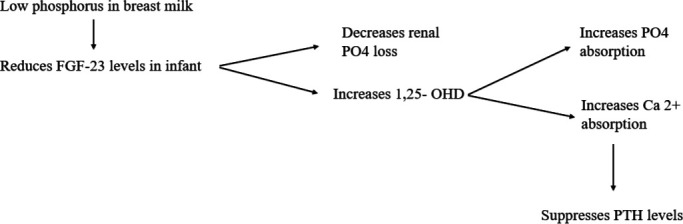
Suggested physiological mechanism for hypercalcaemia in exclusively breastfed infants. Ca, calcium; FGF-23, fibroblast growth factor 23; OHD, 25-hydroxyvitamin D; PO4, phosphate; PTH, parathyroid hormone.

Physiological hypercalcaemia in breastfed infants may be influenced by low phosphate content in breast milk of those mothers, leading to decreased fibroblast growth factor-23 (FGF23) and therefore increased 1,25-dihydroxyvitamin D (1,25OHD). This increased 1,25 OHD in turn improves serum phosphate levels in the infant by increasing phosphate absorption, but also enhancing Ca absorption and suppresses PTH (figure 2).

Future research should measure FGF23, 1,25OHD, Ca and phosphorus content in the breast milk.

### Challenges in breast milk analysis

Accurate measurements of elements in breast milk are technically challenging, as it involves critical stages such as collection, storage, transport and sample preparation, all of which require careful standardisation. Moreover, breast milk samples are first ashed and then digested with a weak acid to break down the sample, and calcium and phosphorus contents are subsequently measured using commercial assay kits on a centrifugal analyser. Measuring the phosphorus content in breast milk is challenging because, without prior ashing and digestion of the sample, only 30%–40% of the total phosphorus content can be detected.^11 12^

### Clinical implications and management

Management of PTH-independent hypercalcaemia in breastfed infants routinely includes discontinuation of breastfeeding, low Ca formula feeds, hyperhydration, bisphosphonates, etc, due to the risk of nephrocalcinosis in conditions such as IIH.^4^ Our findings indicate that breast milk–associated physiological hypercalcaemia is typically asymptomatic and does not result in nephrocalcinosis, supporting a conservative approach to management. Continuation of breastfeeding is considered safe and beneficial in such cases. Nevertheless, careful monitoring is essential throughout the breastfeeding period. We recommend monitoring serum cCa levels every 8–12 weeks if asymptomatic and if initial renal ultrasound findings are normal. Ongoing surveillance also helps ensure that alternative causes of hypercalcaemia are not overlooked.

### Strengths and limitations

This 5- year retrospective natural history study is the first to systematically characterise both biochemical and radiological outcomes in infants with breast milk–associated physiological hypercalcaemia. A major strength of the study lies in its longitudinal follow-up and detailed, consistent data collection.

One study limitation is the lack of maternal clinical information. Owing to the retrospective challenge in data retrieval, maternal medical histories and bone biochemistry data were unavailable. This limited our ability to explore potential maternal contributions to infant hypercalcaemia. The cohort consisted of in-hospital referrals, which may introduce selection bias toward infants with mild confounding factors (eg, transient feeding difficulties or dehydration), although none were clinically symptomatic from hypercalcaemia.

### Future directions

Future studies could include direct measurement of Ca, phosphate and vitamin D metabolites in breast milk to clarify how breast milk composition influences neonatal Ca homeostasis. Parallel evaluation of infant serum 1,25-dihydroxyvitamin D and FGF23 concentrations would add mechanistic understanding of the physiological regulation of Ca and phosphate metabolism in this setting.

## Conclusion

Breast milk–associated physiological hypercalcaemia in asymptomatic infants is a benign and self-limiting condition that does not result in nephrocalcinosis. These findings support a conservative management approach and reinforce the safety of continued breastfeeding without the need for aggressive intervention.

## Supplementary material

10.1136/bmjpo-2025-004230 online supplemental table 1

## Data Availability

Data are available upon reasonable request.
