# Supplementary material for: Breast milk–associated physiological hypercalcaemia: an observational study of clinical, biochemical and radiological outcomes
Source: BMJ Paediatr Open. 2026 May 28;10(1):e004230. doi: 10.1136/bmjpo-2025-004230 (PMC13223625; doi:10.1136/bmjpo-2025-004230)
Supplement: online supplemental table 1 [file bmjpo-10-1-s001.docx]

| Peak serum cCa (mmol/L) | Corresponding Vitamin – D (mmol/L) | Corresponding PTH(pmol/L) | Corresponding PO4 (mmol/L) | Corresponding Mg2+(mmol/L) | Urine calcium : creatinine (mmol/mmol) |
| --- | --- | --- | --- | --- | --- |
| 3.46 | 48 | 1.3 | 2 | 0.95 | 11.7 |
| 3.44 | 64.9 | 0.6 | 1.47 | NA | NA |
| 3.13 | 81.8 | 0.4 | 1.94 | NA | 6.3 |
| 2.92 | 44.9 | 1.3 | 2.17 | 0.77 | 3 |
| 2.87 | 37 | 0.5 | 1.71 | 0.93 | 4.23 |
| 2.84 | 141 | 0.5 | 2.05 | NA | NA |
| 3.05 | 61 | 0.3 | 1.62 | 0.93 | NA |
| 2.93 | 18.9 | 0.3 | 0.89 | NA | 0.75 |
| 3.4 | 40.4 | 0.6 | 1.44 | NA | NA |
| 3.14 | NA | 0.7 | 1.53 | NA | NA |
| 3.2 | 32.1 | 3.5 | 2.18 | NA | 1.12 |
| 3.03 | 41 | 3.3 | 2.91 | NA | 3.33 |
| 2.88 | 20 | 2 | 2.29 | NA | NA |
| 3.29 | NA | 0.5 | 1.59 | 1.05 | 4.54 |
| 2.97 | 32 | 0.5 | 2.09 | 0.83 | 3.43 |
| 3.14 | 95 | 2.3 | 2 | 0.78 | 5.83 |
| 3 | 35 | 1.4 | 2.29 | 0.67 | 1.83 |
| 3.03 | NA | 0.5 | 1.62 | 0.78 | NA |
| 2.85 | 122 | 1 | 1.52 | 0.9 | NA |
| 3.03 | NA | 0.6 | 2.02 | 0.77 | NA |
| 2.95 | 106.1 | 1.7 | 1.89 | 0.95 | NA |
| 3.12 | 74.5 | 0.5 | 2 | 0.77 | 1.6 |
| 3.12 | 74.5 | 0.5 | 2 | 0.77 | 1.6 |
| 3.15 | 63 | 0.5 | 2.28 | NA | NA |
| 3.19 | 22 | 0.6 | NA | NA | NA |

Supplementary table 1 – detailed biochemistry values for each infant. NA (not available)
