# Supplementary material for: Breast milk–associated physiological hypercalcaemia: an observational study of clinical, biochemical and radiological outcomes
Source: BMJ Paediatr Open. 2026 May 28;10(1):e004230. doi: 10.1136/bmjpo-2025-004230 (PMC13223625; doi:10.1136/bmjpo-2025-004230)
Supplement: Supplementary file 2 [file bmjpo-2025-004230.reviewer_comments.pdf]

PEER REVIEW HISTORY

BMJ Paediatrics Open publishes all reviews undertaken for accepted manuscripts. Reviewers are asked to complete a checklist review form and are provided with free text boxes to elaborate on their assessment. These free text comments are reproduced below.

ARTICLE DETAILS

**TITLE (PROVISIONAL)**	Breast milk-associated physiological hypercalcaemia: an observational study of clinical, biochemical and radiological outcomes.
**AUTHORS**	Govindarajan, Sandhya<br>Zulf Mughal, Mohamed<br>Zamir, Imran<br>Padidela, Raja<br>Chinoy, Amish

VERSION 1 – REVIEW

**REVIEWER NAME**	Reviewer 1
**REVIEWER AFFILIATION**	
**REVIEWER CONFLICT OF INTEREST**	No Conflict of Interest
**AI DISCLOSURE**	No, I have not used AI
**DATE REVIEW RETURNED**	12-Sep-2025

**GENERAL COMMENTS**	Comments not published pending reviewer's consent

VERSION 2 – REVIEW

**REVIEWER NAME**	Chesover, Alexander
**REVIEWER AFFILIATION**	
**REVIEWER CONFLICT OF INTEREST**	No Conflict of Interest
**AI DISCLOSURE**	No, I have not used AI
**DATE REVIEW RETURNED**	09-Mar-2026

**GENERAL COMMENTS**	This is a clearly written manuscript with a practical and focussed key message. The previous review's major and minor comments have been addressed and answered. This phenomenon is now better clarified as physiological, and its presentation is distinguished from a likely pathologically aetiology that would require additional work-up and management. There is valuable additional data presented in the Tables. There is more clarity in the methods and limitations. No further suggested changes.

VERSION 2 – AUTHOR RESPONSE

Reviewer: 1

Alexander Chesover

Comments to the Author

This is a clearly written manuscript with a practical and focussed key message. The previous review's major and minor comments have been addressed and answered. This phenomenon is now better clarified as physiological, and its presentation is distinguished from a likely pathologically aetiology that would require additional work-up and management. There is valuable additional data presented in the Tables. There is more clarity in the methods and limitations. No further suggested changes.

Reviewer: 1

If you have selected 'Yes' above, please provide details of any competing interests.: Not applicable
